# Germline *POT1* variants are associated with long telomeres and an unexpected broad spectrum of malignancies

**DOI:** 10.1016/j.gimo.2026.104483

**Published:** 2026-07-05

**Authors:** Aasem Abu Shtaya, Shahar Aloni, Marina Eskin-Schwartz, Nethanel Asher, Noy Azulay, Lina Basel-Salmon, Lily Bazak, Rinat Bernstein Molho, Liza Douiev, Sarit Farage Barhom, Eitan Friedman, Ofer Isakov, Ahmad Mahamid, Orli Michaeli, Inbal Kedar, Zohar Levi, Sari Liberman, Shikma Mordechai, Ofer Reiter, Gili Reznick Levi, Shiri Shkedi Rafid, Zinab Sarsor, Orit Uziel, Sharon Simchoni, Tom Walsh, Ofir Wolach, Ori Segol, Tamar Yablonski-Peretz, Aviad Zick, Avinoam Gluck, Yehuda Tzfati, Yael Goldberg

**Affiliations:** 1 Recanati Genetics Institute, Rabin Medical Center – Beilinson Hospital, Petah Tikva, Israel; 2 Unit of Gastroenterology, Lady Davis Carmel Medical Center, Haifa, Israel; 3 Genetics Institute, Soroka University Medical Center, Beer Sheva, Israel; 4 Faculty of Health Sciences, Ben-Gurion University of the Negev, Beer Sheva, Israel; 5 Melanoma and Skin Cancer Center at the Davidoff Center, Rabin Medical Center, Petah Tikva, Israel; 6 Gray Faculty of Medical and Health Sciences, Tel Aviv University, Tel Aviv, Israel; 7 Medical Oncology Department, Chaim Sheba Medical Center, Ramat-Gan, Israel; 8 Department of Genetics, Hadassah Medical Center, Jerusalem, Israel; 9 Faculty of Medicine, Hebrew University, Jerusalem, Israel; 10 Meirav High Risk Clinic, Chaim Sheba Medical Center, Tel-Hashomer, and the Center for Personalized Preventive Medicine, Assuta Medical Center, Tel Aviv, Israel; 11 Clalit Research Institute, Innovation Division, Clalit Health Services, Ramat Gan, Israel; 12 Division of Surgery, Carmel Medical Center, Haifa, Israel; 13 The Ruth and Bruce Rappaport Faculty of Medicine, Technion, Israel Institute of Technology, Haifa, Israel; 14 Pediatric Hematology-Oncology, Schneider Children’s Medical Center of Israel, Petah Tikva, Israel; 15 Division of Gastroenterology, Rabin Medical Center-Beilinson Hospital, Petah Tikva, Israel; 16 Medical Genetics Institute, Shaare Zedek Medical Center, Jerusalem, Israel; 17 Genetic Unit, Ziv Medical Center, Safed, Israel; 18 Division of Dermatology, Rabin Medical Center, Petah Tikva, Israel; 19 The Genetics Institute, Rambam Health Care Campus, Haifa, Israel; 20 The Felsenstein Medical Research Center, Petah Tikva, Israel; 21 Institute of Hematology, Davidoff Cancer Center, Petah Tikva, Israel; 22 The Genetics Institute and Genomics Center, Tel Aviv Sourasky Medical Center, Tel Aviv, Israel; 23 Department of Medicine, University of Washington, Seattle, WA; 24 Department of Genome Sciences, University of Washington, Seattle, WA; 25 Departement of Oncology, Sharett Intitute of Oncology, Hadassah Medical Center, Jerusalem, Israel; 26 Department of Genetics, The Silberman Institute of Life Sciences, The Hebrew University of Jerusalem, Jerusalem, Israel

**Keywords:** Cancer predisposition syndrome, Founder variant, Melanoma, *POT1*, Telomere

## Abstract

**Purpose:**

This study aimed to expand the clinical and biological understanding of *POT1* tumor predisposition syndrome and raise awareness of its broad tumor spectrum and association with ultralong telomeres.

**Methods:**

We conducted a retrospective analysis of 44 individuals carrying pathogenic or likely pathogenic *POT1* variants identified between 2018 and 2025. Clinical, pathologic, and demographic data were extracted from medical records. Telomere length was assessed using in-gel hybridization of telomeric restriction fragments.

**Results:**

Of 44 heterozygotes, 31 had the c.233T>C; p.(Ile78Thr) variant, 8 had c.1672dup; p.(Tyr558Leufs∗6), and 5 had other frameshifting, likely loss-of-function variants. Cancer was diagnosed in 27 heterozygotes (61%), totaling 84 primary malignancies (range: 0-8 per person), with most cases (79%) occurring after the age of 50 years. Melanoma (34%) and breast cancer in females (45%) were the most common. Additional tumors included papillary thyroid carcinoma, desmoid tumors, and diverse malignant and benign neoplasms. Colonic polyps were frequent. *POT1* heterozygotes with the p.(Ile78Thr) variant exhibited significantly longer telomeres than control participants, with a 62-year-old woman showing comparable or even longer telomeres than her children.

**Conclusion:**

*POT1* pathogenic variants confer susceptibility to a wide tumor spectrum, notably melanoma and, unexpectedly, breast cancer. At least some of these variants are linked to ultralong telomeres, consistent with findings from this study and others. These findings support incorporating *POT1* into multigene panels and suggest telomere length as a potential biomarker for diagnosis.

## Introduction

Telomeres, the protective caps at the ends of chromosomes, are essential for preserving genomic integrity and regulating cellular proliferation.^1^^,^^2^ They serve a dual role in cancer prevention by (1) maintaining genomic stability by shielding chromosomal ends from recognition by the DNA damage response machinery and (2) imposing a replicative limit on cell division by progressively shortening with age. Although the pathologic consequences of telomere shortening are well established, the impact of excessively long telomeres on cancer susceptibility remains elusive.^3^ Shelterin is a 6-protein complex that acts as a protective cap and regulates telomere length and interactions. POT1, a key component of Shelterin, binds single-stranded telomeric DNA to safeguard chromosome ends from aberrant DNA damage response activation and to regulate telomere length.^4^ Germline heterozygous pathogenic variants (PVs) in *POT1* (HGNC:17284, HG38: chr7:124822385-124929825) cause *POT1*-tumor predisposition syndrome (*POT1*-TPDS, OMIM 615848), which is often, but not always, associated with abnormally long telomeres.^5^, ^6^, ^7^ Initially, *POT1*-TPDS was linked mainly to chronic lymphocytic leukemia (CLL), melanoma, and angiosarcoma.^7^^,^^8^ The associated phenotype has been broadened to include a diverse range of benign and malignant neoplasms involving epithelial, mesenchymal, and neuronal tissues, as well as lymphoid and myeloid malignancies.^5^^,^^9^ In addition, *POT1* germline PVs or likely PVs have been associated with clonal hematopoiesis in an autosomal dominant inheritance pattern.^5^^,^^6^ Given the small number of heterozygotes described, the true prevalence of *POT1*-TPDS remains undetermined. Most reports were limited by the small number of heterozygotes; therefore, the overall lifetime cancer risks are unclear, and data on long-term follow-up of identified families are limited. Founder *POT1* variants have been identified in specific populations, including Ashkenazi Jews (AJ) and Italians,^10^ providing opportunities to obtain more data and explore genotype-phenotype correlations across genetically distinct groups while suggesting common pathogenic mechanisms.

Recent efforts have focused on establishing evidence-based clinical management. The UK’s clinical practice guidelines represent the first systematic attempt at making surveillance recommendations; however, they focus mainly on CLL, angiosarcoma, melanoma, and gliomas, emphasizing the need for further research to refine risk stratification and optimize care.^11^ Here, we present a large cohort of heterozygotes for *POT1* PVs, describe their associated clinical phenotypes, and assess their telomere length. These findings have direct implications for genetic counseling and surveillance protocols and support the inclusion of *POT1* in multigene cancer predisposition panels. They also highlight the potential importance of measuring telomere length in cancer risk assessment.

## Materials and Methods

### Study population and data collection

We conducted a retrospective analysis of 44 individuals carrying *POT1* PVs, identified between 2018 and 2025. Referral indications for genetic testing varied and included personal or family history of cancer, multiple benign tumors, *POT1* PVs detected during tumor profiling, or cascade testing following detection of a familial variant. Two patients were referred for oncogenetic counseling following the incidental identification of *POT1* variants during trio-exome sequencing that was performed for developmental delay in their children. Relevant information was extracted from medical records. Clinical and demographic information collected included data on age, sex, ethnicity, tumor features, and detailed personal and family histories.

### Searching for genetic variants

Germline genetic testing was carried out for clinical purposes using one of the following platforms: cascade testing for familial variants or multigene cancer predisposition panels, including the Invitae Multi-Cancer Panel–84 genes (Invitae Corp), Blueprint Comprehensive Hereditary Cancer Panel–160 genes (Blueprint Genetics), cancer panels at the Genomic Center of the Genetic Institute at Tel Aviv Sourasky Medical Center–92 genes and at Clalit Genomic Center–187 genes; or exome sequencing at CeGat Laboratory (CeGaT GmbH) or at Hadassah Medical Center. Somatic genotyping was done using the Oncomine Comprehensive Assay Plus (Thermo Fisher Scientific) in DNA and RNA extracted from formalin-fixed paraffin-embedded tissues. Variant classification was done according to the American College of Medical Genetics and Genomics guidelines.^12^ Annotation was based on the Matched Annotation from the NCBI and EMBL-EBI (MANE) Select transcript NM_015450.3, with genomic coordinates aligned to GRCh37/hg19 (RefSeq genome assembly accession number: GCF_000001405.25).

### Genomic DNA extraction

The genomic DNA obtained from the submitted samples was processed for sequencing as described.^9^ Different laboratories used various methods for the identification of sequence variants in the aligned reads. All laboratories had processes in place for detecting copy number variations. For measuring telomere length, a portion of each blood sample was separated into granulocyte and mononuclear fractions by standard Ficoll gradient centrifugation. Another portion was treated with red blood cell lysis buffer as described,^13^ yielding an additional fraction of whole blood leukocytes. Genomic DNA was extracted from the 3 fractions of each sample by standard proteinase K/phenol extraction and ethanol precipitation.

### In-gel analysis of telomeric restriction fragments

Genomic DNA samples were digested with the HinfI restriction endonuclease and quantified by a Qubit 4 fluorometer (Thermo Fischer Scientific Inc). Equal amounts (1-5 μg) of the digested DNA samples were separated on a 0.6% agarose gel in 1 × Tris-Borate-EDTA buffer at 1600 V for 1 hour. Gels were ethidium-stained, dried, prehybridized for 30 to 60 minutes at 50 °C in the hybridization solution (sodium phosphate buffer [0.5 N Na^+^, pH 7.2], 1 mM EDTA, and 7% sodium dodecyl sulfate[weight/volume]), and hybridized overnight at 50 °C in hybridization solution with a probe mixture containing end-labeled telomeric oligonucleotide (AACCCT)_3_ and 1 kb Extend DNA Ladder (NEB Inc). Ladder probes were digested with HinfI, dephosphorylated by quick calf intestinal phosphatase (NEB Inc), and heat-inactivated before labeling. All probes were 5′ end-labeled with (γ-^32^P)- adenosine triphosphate and T4 polynucleotide kinase (NEB Inc). After hybridization, the gels were washed (for 10, 30, and 50 minutes) with 4 × saline sodium citrate buffer at room temperature and once more with prewarmed 4 × saline sodium citrate buffer at 45 to 50 °C for 30 minutes. The gels were exposed to a Typhoon FLA 9500 biomolecular Imager (GE Healthcare Life Sciences). The mean length of the telomeric restriction fragments (TRFs) was quantified using WALTER.^14^ For each sample, we analyzed 3 fractions: granulocytes, peripheral blood mononuclear cells (PBMCs), and whole blood leukocytes.

## Results

### Cohort characteristics and variant spectrum

Overall, 44 individuals from 31 seemingly unrelated families who carried *POT1* PVs were included: 15 were males (34%), and 29 were females (66%), with an average age of 55 years (range: 15-82) and a median age of 55 years. Most participants (33/44, 75%) were of AJ ancestry (Table 1).

**Table 1:** Demographic characteristics of *POT1* pathogenic variant heterozygotes (*N* = 44)

Characteristics	Age, Number and Percentage (in parenthesis) of the Participants in the Study
**Gender**	
Female	29 (66%)
Male	15 (34%)
**Age, y**	
Average age	55
Median age (range)	55 (15-82)
**Age distribution**	
<30	4 (9%)
30-50	11 (25%)
>50	29 (66%)
**Ethnicity**	
Ashkenazi Jewish	33 (75%)
Tunisian Jewish	8 (18%)
Mixed or other	3 (7%)

Values are presented as *n* (%), unless otherwise specified.

### *POT1* variant spectrum

**Figure 1:**
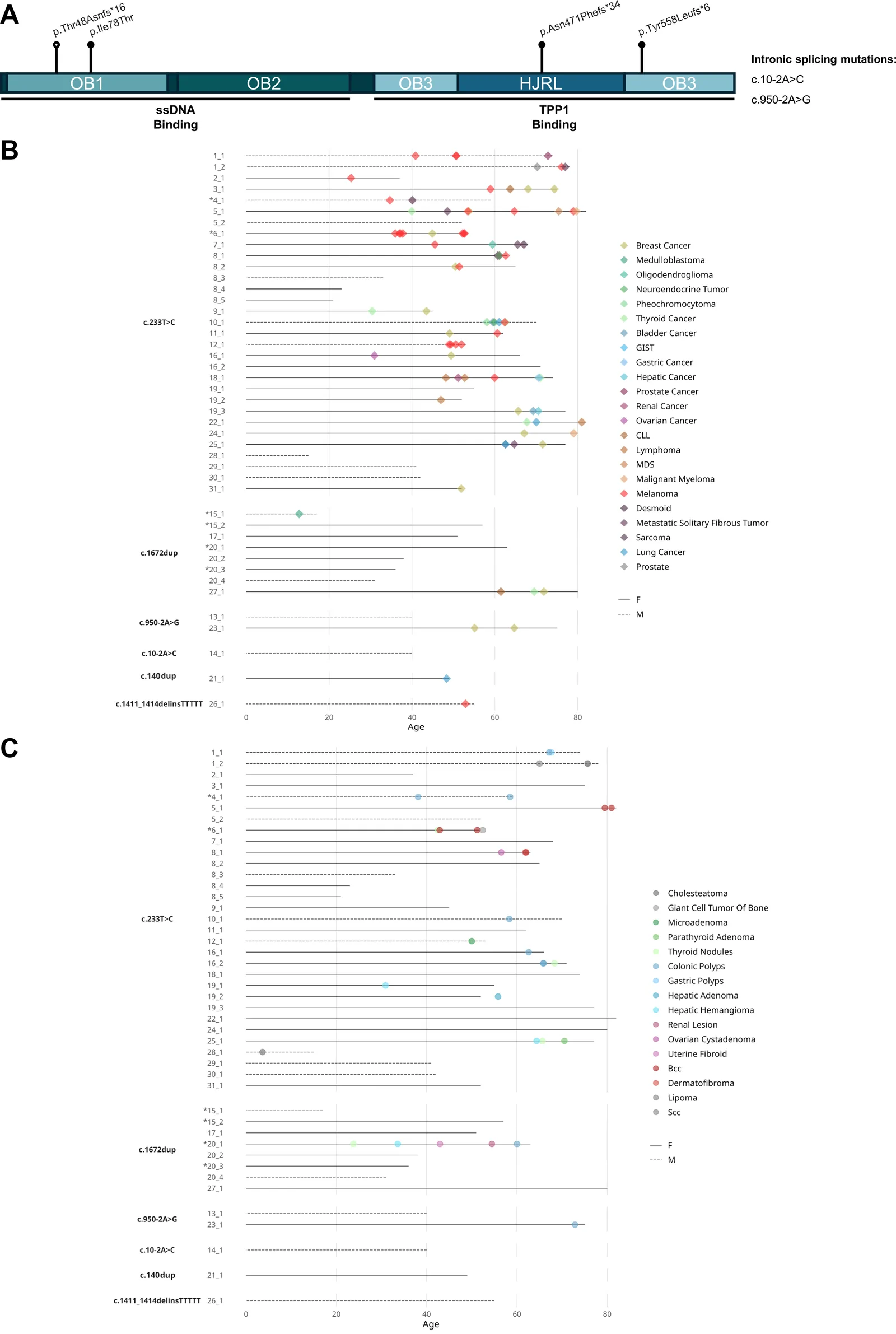
***POT1*variants.** (A) Structural organization of POT1 protein domains. Missense (full circle) and frameshifting (empty circles) pathogenic variants (PVs) are indicated along the protein sequence, and intronic splicing PVs are indicated below. DNA and protein interactions are denoted below the scheme. Indicated are oligonucleotide/oligosaccharide-binding (OB) fold domains 1 to 3 and Holliday junction resolvase-like domain (HJRL). The onset age of malignant (B) and benign (C) tumors among *POT1* heterozygotes is presented schematically. Patients (denoted as <family number>_<patient number>) are stratified by *POT1* variant. Patients with an additional PV in other cancer predisposition genes are marked by “∗”. Bcc, basal cell carcinoma; CLL, chronic lymphocytic leukemia; F, female; GIST, gastrointestinal stromal tumor; M, male; MDS, myelodysplastic syndromes; Scc, squamous cell carcinoma.

Six distinct PVs were identified across the cohort, comprising 1 missense, 2 splice-site, and 3 insertion/deletion variants (Figure 1A, Table 2). PVs were distributed across key domains of POT1, including oligonucleotide/oligosaccharide-binding fold domains 1 (OB1) and 3 and the C-terminal TPP1-binding domain. The most frequent variant was the AJ founder missense PV c.233T>C; p.(Ile78Thr), observed in 31 of 44 heterozygotes (70%). The second most common was the frameshift variant c.1672dup; p.(Tyr558Leufs∗6), identified in 8 individuals (18%). Additional variants included 2 splicing variants (c.950-2A>G, 2/44 [4.55%] heterozygotes; c.10-2A>C, 1/44 [2.27%] heterozygotes) and 2 more indels (c.140dup and c.1411_1414delinsTTTTT, 1/44 [2.27%] heterozygote each). The AJ founder missense PV affects a highly conserved residue in OB1 and compromises single-stranded telomeric DNA binding. The frameshift variants likely result in loss of function through premature termination and loss of critical functional domains, as well as degradation by the nonsense-mediated decay pathway. Notably, 6 individuals harbored an additional monoallelic PV in other cancer predisposition genes: *MSH6* c.1168_1170delinsAA p.(Asp390Asnfs∗21) (heterozygotes 20-1 and 20-3); *MITF* c.1273G>A p.(Glu425Lys) (6-1); *RECQL4* c.1166_ 1168Del p.(Cys389del) (15-1 and 15-2); and *BLM* c.2207_2212delinsTAGATTC p.(Tyr736Leufs∗5) (4-1) (Figure 1).

**Table 2:** The spectrum of *POT1* pathogenic variants

DNA change	Protein Change	Genomic location (assembly: GRCh38)	Type of Variant	Number of Patients
NM_015450.3:c.233T>C	p.(Ile78Thr)	NC_000007.14:g.124870933A>G	Missense	31
NM_015450.3:c.1672dup	p.(Tyr558Leufs∗6)	NC_000007.14:g.124827228dup	Indel	8
NM_015450.3:c.950-2A>G		NC_000007.14:g.124847000T>C	Splice	2
NM_015450.3:c.10-2A>C		NC_000007.14:g.124892382T>G	Splice	1
NM_015450.3:c.140dup	p.(Thr48Asnfs∗16)	NC_000007.14:g.124871026dup	Indel	1
NM_015450.3:c.1411_1414delinsTTTTT	p.(Asn471Phefs∗34)	NC_000007.14:g.124835370_124835373delinsAAAAA	Indel	1

### Clinical features and cancer phenotype

The 44 *POT1* variant heterozygotes exhibited a total of 84 primary malignancies, diagnosed between the ages of 12 and 80 years, with an average of 1.9 tumors per heterozygote (range: 0-8). Age-related tumor occurrence and spectrum in each of the heterozygotes are visualized in Figure 1B. A total of 27 individuals (61%), comprising 20 females and 7 males, had at least 1 malignancy; 14% of heterozygotes had a single malignancy, 16% had 2, and 32% had 3 or more primary tumors. Notably, 4 individuals were affected by more than 5 tumors. Among 29 heterozygotes aged ≥50 years, 23 (79%) had cancer, and 20 (69%) developed at least 2 primary malignancies. Females had an average of 2.2 cancers per person compared with 1.4 cancers per person in males. The median age at diagnosis was 49 years (range: 12-70) for the first malignancy among heterozygotes. Early-onset cancer (before the age of 20 years) was observed in a single individual (15-1); one additional heterozygote (2-1) had cancer diagnosed before the age of 30 years. By the age of 40 years, 8 of 27 affected heterozygotes (30%) had developed their first malignancy, and by the age of 50 years, 14 of 27 (52%) were affected. The remaining 13 of 27 individuals (48%) were initially diagnosed with cancer after the age of 50 years.

### Cancer types

The most frequent cancer noted was cutaneous melanoma, diagnosed in 15 of 44 individuals (34%). Of these, 14 individuals (93%) carried the variant c.233T>C. The average age at the first melanoma diagnosis was 51.6 years (range: 25-79). Four heterozygotes developed recurrent melanomas at multiple body sites (3-7 distinct events). Multiple nevi were reported in 8 of the 15 patients with melanoma. Melanoma frequently co-occurred with other tumor types, including breast cancer (*n* = 5), papillary thyroid cancer (PTC) (*n* = 3), and desmoid tumors (*n* = 2). In 6 of 12 individuals (50%) with multiple tumors who also had melanoma, melanoma was the first malignancy diagnosed (Figure 1B). Among the 29 female heterozygotes, 13 (45%) were diagnosed with breast cancer (between the ages of 44 and 74 years); in 4 (31%), breast cancer had occurred before the age of 50 years; in 4, breast cancer occurred between the ages of 50 and 59 years; and in 5, breast cancer occurred between the ages of 60 and 74 years. Two women had bilateral breast cancer. Five women (17%) had both melanoma and breast cancer. Breast cancer was the initial malignancy in 5 of 16 women (31%) with multiple tumors (Figure 1B). PTC was reported in 5 patients (aged 31-71 years), all of whom had at least 1 additional primary tumor. In 4 of 5 heterozygotes, PTC was the first tumor to occur (Figure 1B, Table 3). Of the “traditional” *POT1*-TPDS tumors, CLL was reported only in 1 heterozygote (aged 62). Sarcoma was reported in 3 individuals, and brain tumors were reported in 2 individuals. Tumor molecular profiling was available for only 2 samples of lung cancer. Both cases revealed microsatellite-stable status, with low tumor mutational burdens of 7.6 and 3.8 somatic mutations/Mb, respectively. The variant allele frequency (VAF) for the *POT1* germline variant was 50% in both tumors, without evidence of a somatic second hit.

**Table 3:** Most common cancer types in all *POT1* pathogenic variant heterozygotes (*N* = 44)

Cancer Type	Frequency	% Heterozygotes	Age at Diagnosis, y
Melanoma	15	34%	52 (25-79)
Breast cancer	13	45%^a^	59 (44-74)
Hematologic malignancies (CLL, MDS, MM, Hodgkin, and NHL)	7	16 %	65 (47-80)
Papillary thyroid carcinoma	5	11%	55 (31-71)
Desmoid	3	7%	55 (41-66)
Sarcomas	3	7%	55 (48-65)
Lung cancer	3	7%	60 (48-70)
Renal cell carcinoma	2	5%	57 (52-62)
Neuroendocrine tumor	2	5%	61 (60-62)
Brain tumors	2	5%	36 (12-60)

Values are presented as *n* or % or average (range).

*CLL*, chronic lymphocytic leukemia; *MDS*, myelodysplastic syndromes; *MM*, multiple myeloma; *NHL*, non-Hodgkin lymphoma.

a % of 29 females.

### Benign tumors

A total of 34 benign tumors were documented in 16 heterozygotes (Figure 1C). The most frequently reported were colonic polyps, identified in 7 individuals (ages 39-73 years), with a mean age of 61 years at the first polyp detection. Additional benign findings included thyroid nodules (*n* = 4) and hepatic lesions (*n* = 4). Two of the c.1672dup variant heterozygotes, a mother and a daughter, also coinherited Lynch syndrome; the mother had a few colonic polyps at the age of 60 years and no malignancy (Figure 1C).

### Telomere length

**Figure 2:**
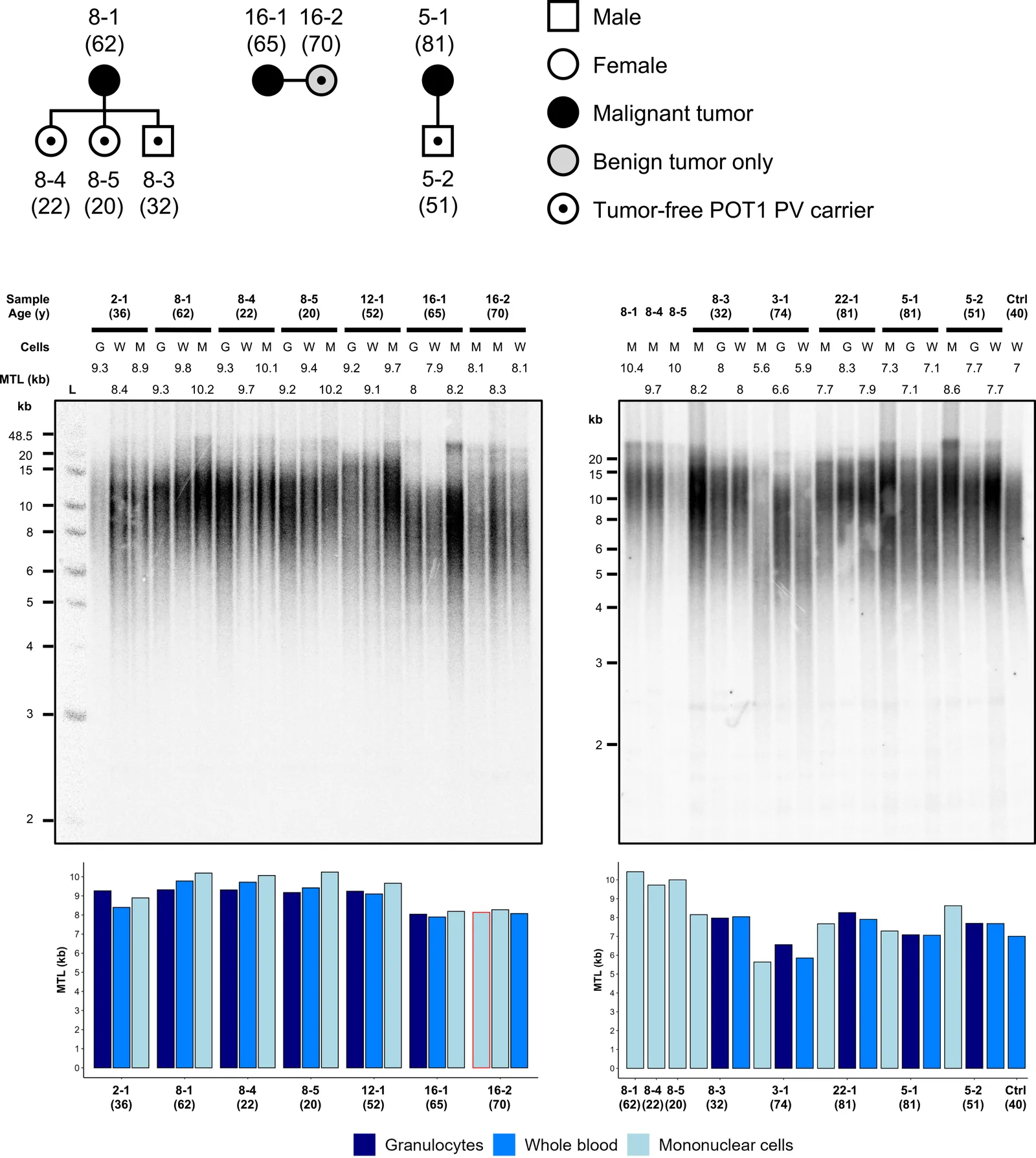
**Long telomeres in *POT1*^I78T^ heterozygotes.** Genomic DNA was extracted from whole blood (W), granulocytes (G), or peripheral blood mononuclear cells (M) of 12 *POT1*^I78T^ heterozygotes from 7 unrelated families (family trees are shown on top) and a healthy unrelated individual, digested with HinfI endonuclease, electrophoresed, and hybridized with a telomeric probe, as described in the Methods section. Samples 8-1 (M), 8-4 (M), and 8-5 (M) are included in both gels. Mean telomeric restriction fragment length (MTL) values were calculated by WALTER,^14^ cell type and ages are indicated above the lanes. MTL values are plotted below. All DNA samples were extracted by the standard proteinase K/phenol procedure. The sample 16-2 (M) was split and extracted by both Monarch genomic DNA purification kit (NEB Inc; outlined in red) and proteinase K/phenol (on its right), yielding similar MTLs. PV, pathogenic variant.

Telomere length was evaluated in the blood of 12 *POT1* c.233T>C; p.(Ile78Thr) heterozygotes belonging to 7 families and in a healthy unrelated 40-year-old individual. As shown in Figure 2, 11 of the 12 heterozygotes displayed higher mean TRF length (MTL) in whole blood leukocytes than was measured for the control (MTL = 7 kb). Even the oldest patients (individuals 22-1 and 5-1), aged 81 years, had a higher or comparable whole blood leukocyte MTL (7.9 and 7.1 kb, respectively) than the control, despite being 41 years older. The only heterozygote (3-1) with a lower MTL (5.9 kb in whole blood leukocytes) was also diagnosed with CLL, which could explain the shortened telomeres due to excessive cell proliferation, as reported previously.^15^ PBMCs displayed longer telomeres than granulocytes in most samples (Figure 2). The mother in family 8 (individual 8-1, age 62 years) showed comparable telomere length to her 2 children who were also heterozygotes (individuals 8-4 and 8-5, aged 22 and 20 years, respectively) and longer telomeres than her other sibling (individual 8-3, age 32 years): in PBMCs, MTL was 10.2 kb for individual 8-1, 10.1 kb for individual 8-4, and 10.2 kb for individual 8-5; and in whole blood leukocytes, MTL was 9.8 kb for individual 8-1, 9.7 kb for individual 8-4, and 9.4 kb for individual 8-5 (Figure 2, left). This trend was recapitulated in the second in-gel experiment (Figure 2, right), where the MTL in PBMCs was 10.4 kb for individual 8-1, 9.7 kb for individual 8-4, and 10 kb for individual 8-5, with a third heterozygote child (individual 8-3) having an even shorter MTL of 8.2 kb in PBMCs. This is unusual because lymphocyte telomeres shorten with age in the general population at an average rate of 43 bp per year, and thus, the mother is expected to have telomeres shorter by about 1.7-1.8 kb than her children who are 40 and 42 years younger (individuals 8-4 and 8-5, respectively), and 1.3 kb shorter than individual 8-3, who is 30 years younger.^16^ Instead, in the extreme case, she displayed 2.2 kb longer telomeres than her son (individual 8-3). This observation suggests that the telomeres of proband 8-1 do not shorten with age as much as they do in the general population and thus increasingly deviate with age from the average telomere length in their age group.

## Discussion

This large cohort of 44 individuals with germline *POT1* PVs broadens the clinical and phenotypic spectrum of *POT1*-TPDS and provides novel data on telomere length among heterozygotes. Our findings reinforce the role of POT1 in preserving genomic stability across multiple tissues and have important implications for genetic counseling, surveillance strategies, and clinical management.

### Tumor spectrum and cancer risk

The malignancies observed span multiple tissue types, perhaps reflecting the fundamental role of telomere dysfunction in diverse cellular contexts. The observed malignancy spectrum significantly expands previously reported associations with *POT1* beyond melanoma, glioma, thyroid, and hematologic malignancies to include breast, desmoid, and other solid tumors, as well as various benign tumors, including colonic polyps. The mean number of malignancies per heterozygote was 1.9, increasing with age to 2.7 among individuals older than 50 years, with no significant difference between sexes in this age group. Fewer than 10% of heterozygotes were diagnosed before the age of 30 years, whereas more than 50% had developed at least 1 malignancy by the age of 50 years, supporting an age-dependent penetrance model. We could not find any association between the burden of benign tumors and malignancies. For example, patient 20-1 had 5 different benign tumors by the age of 62 years but was cancer-free, and patient 5-1 had 8 primary cancers but only 2 benign tumors. Melanoma was diagnosed in 34% of heterozygotes, consistent with prior reports of *POT1* as a melanoma predisposition gene.^17^, ^18^, ^19^, ^20^ Notably, 45% of female heterozygotes developed breast cancer (age range: 44-74 years), suggesting that breast cancer is a more integral component of *POT1*-TPDS than previously recognized. These findings justify including breast imaging in surveillance protocols for female heterozygotes no later than the age of 40 years and testing for *POT1* in women diagnosed with breast cancer. PTC and desmoid tumors have been reported in a subset of this cohort.^9^ Here, PTC was reported in 5 patients (12%), aligning with previous reports of PVs in telomere-machinery genes (*POT1*, *TINF2*, and *ACD/TPP1*) in 4.5% of familial PTC cases and 1.5% of unselected cases.^5^ Desmoid tumors were identified in 3 individuals (ages 40, 60, and 66 years); all of them were either intra-abdominal or within the abdominal wall, highly aggressive, and recurrent. None of the individuals with desmoid tumors carried an *APC* PV or had a history of abdominal trauma or surgery. Tumor genomic profiling was available for 2 lung malignancies; both tumors exhibited microsatellite stability, low tumor mutational burden, and no loss of heterozygosity at the *POT1* locus. These limited somatic data represent the first description of *POT1*-associated tumor characteristics and may offer preliminary insight into underlying pathogenesis.

### Genotype-phenotype correlations

The recurrent missense variant c.233T>C; p.(Ile78Thr), present in approximately 70% of heterozygotes, was associated with a higher tumor burden (mean: 2.43 malignancies) and more than 90% abundance of melanoma. This substitution of a highly conserved residue within the OB1 domain, which is essential for telomeric single-stranded DNA binding, was shown to compromise POT1 function.^6^^,^^7^^,^^21^, ^22^, ^23^ Six individuals carried a second PV in a cancer predisposition gene. One notable case (individual 6-1) who also carried the *MITF*:c.1273G>A; p.(Glu425Lys) variant associated with a higher risk for melanoma and developed 7 primary melanomas. Thus, the 2 PVs may have an additive effect on the risk of developing melanoma. Interestingly, the 2 double heterozygotes for *MSH6* and *POT1* did not present with Lynch syndrome–associated morbidity. Because some telomere shortening has been observed in patients with Lynch syndrome,^24^^,^^25^ it would be interesting to study the genetic interactions between these variants in the double heterozygotes and whether the *MSH6* and *POT1* variants could counterbalance their effects on telomeres and compensate for each other. The phenotypic impact of dual PVs remains uncertain and warrants further investigation.

### Telomere dysfunction and age-related morbidity

The progressive increase in tumor burden with age suggests a cumulative effect over time, possibly because of telomere dysfunction. In cases of malignancies driven by short telomeres, gradual telomere attrition, particularly in tissues with high proliferative demands or environmental exposures, leads to genomic instability, which in turn causes malignant transformation. In the case of the AJ missense founder PV in *POT1* described here, p.(Ile78Thr), the lack of age-dependent shortening and the longer telomeres than those of an age-matched unrelated healthy control suggest that the oncogenicity of this *POT1* PV is due to a different mechanism. It has been suggested that longer telomeres confer greater proliferative potential and thus a greater chance for the accumulation of other oncogenic variants.^6^ POT1 dysfunction may also induce replicative stress and increased accumulation of DNA damage at telomeres.^7^^,^^26^ Because telomeres are hard to replicate because of their repetitive guanine– and cytosine–rich nature, the longer the telomeres are, the more replication stress and telomeric damage can be expected. PBMCs displayed longer telomeres than granulocytes in most samples (Figure 2), suggesting that PBMCs (which are mostly composed of lymphocytes) elongate their telomeres more than granulocytes, either because they divide more or because of a higher telomerase expression level.

### Clinical management implications

These findings support a need for broad surveillance in *POT1* heterozygotes. The recently published UK clinical guidelines recommend dermatologic surveillance from the age of 18 years and cardiac magnetic resonance imaging in those with a family history of cardiac angiosarcoma.^11^ Based on our data, we endorse dermatologic screening and education for melanoma risk, breast imaging for women beginning no later than the age of 40 years, and consideration of thyroid ultrasound and periodic complete blood counts for the early detection of hematologic malignancies. Although c.233T>C heterozygotes had a higher tumor burden, current evidence does not yet justify genotype-specific surveillance protocols.

### Study limitations and future directions

This study’s retrospective design and possible ascertainment bias may limit the generalizability of the findings. The small number of individuals carrying nonrecurrent variants and additional PVs restricts the interpretation of variant-specific phenotypes. In addition, apparent sex differences can arise from clinic-based ascertainment bias rather than true biology; an adjusted analysis excluding sex-specific tumors did not reveal any evidence that penetrance is modified by sex. Future studies should prioritize prospective, multicenter cohort designs, as well as functional and molecular analyses of POT1 variant proteins to better understand the mechanisms involved in cancer predisposition.

Establishing national and international registries of *POT1* PV heterozygotes, with linkage to cancer registries and telomere biology data for additional *POT1* PV variants, is important to determine whether long telomeres are typical of all patients with *POT1*-TPDS, suggesting a common mechanism. This understanding will be critical for using telomere length as a biomarker, refining cancer risk estimates, and understanding genotype-phenotype correlations. Further studies are needed to elucidate the role of telomere dynamics in cancer predisposition. As telomere-targeted therapies emerge, an improved understanding of *POT1*-driven oncogenesis may open new avenues for intervention. Our findings contribute to the evolving precision medicine framework for hereditary cancer syndromes in which genetic information informs individualized care and surveillance strategies.

## Data Availability

Additional data that support the findings of this study are available from the corresponding authors (Y.G. or Y.T.) upon request.

## Ethics Declaration

The study was conducted at Rabin Medical Center, Hadassah Medical Center and telomere analysis at the Genetics department, the Hebrew University. The study was approved by the ethics committees of Rabin and Hadassah Medical Centers (approvals RMC-no. 0847-22 and HMC-no. 0339-20, respectively). Informed consent was obtained from participants in accordance with institutional ethical standards. All human studies adhered to the principles set out in the Declaration of Helsinki. Data were de-identified for analysis.

## Declaration of Generative AI and AI-Assisted Technologies in the Writing Process

During the preparation of this work, the author(s) used ChatGPT in order to improve language, readability, and formatting. After using this tool, the author(s) reviewed and edited the content as needed and take(s) full responsibility for the content of the publication.

## Conflict of Interest

The authors declare no conflicts of interest.
